# Grades of Poorly Differentiated Clusters are Associated with Lymph Node and the Tumour, Node and Metastasis Stages in Colorectal Carcinoma

**DOI:** 10.21315/mjms2023.30.6.8

**Published:** 2023-12-19

**Authors:** Thanamogan Kerisnon@Krishnan, Norhafizah Mohtarrudin, Wan Azura Wan Yaacob, Huzlinda Hussin

**Affiliations:** 1 Department of Pathology, Hospital Raja Permaisuri Bainun, Perak, Malaysia; 2 Department of Pathology, Faculty of Medicine and Health Sciences, Universiti Putra Malaysia, Selangor, Malaysia; 3 Department of Pathology, Hospital Serdang, Selangor, Malaysia

**Keywords:** colorectal carcinoma, tumour grading, prognostic factors, cell differentiation

## Abstract

**Background:**

Colorectal carcinoma (CRC) is the third most common cancer globally. In Malaysia, CRC is most prevalent among males and the second most common cancer among females. The CRC arises mainly from the adenocarcinoma sequence. Poorly differentiated clusters (PDCs) and tumour budding (TB) are believed to represent sequential steps in tumour growth. Therefore, this study analysed the association between PDC grades with clinicopathological and demographic characteristics of CRC.

**Methods:**

A total of 47 CRC cases previously diagnosed by histopathological examination were reviewed for the presence of PDCs and graded accordingly. The association between PDC grades with clinicopathological and demographic characteristics was statistically analysed.

**Results:**

Out of the 47 cases with PDCs, most of them were of grade 3 (G3) (*n* = 27, 57.4%), followed by grade 2 (G2) (*n* = 13, 27.7%) and grade 1 (G1) (*n* = 7, 14.9%). Higher PDC grades (G2 and G3) were mainly observed in higher tumour stage (T); T3 (*n* = 26, 83.9%), T4 (*n* = 12, 92.3%), N1 (*n* = 20, 86.9%), N2 (*n* = 15, 100%). In addition, there was a significant association between PDC grades with the nodal stage (N) (*P* = 0.013) and the tumour, node and metastasis (TNM) stages (*P* = 0.012).

**Conclusion:**

The PDC grades are useful for assessing the disease prognosis in CRC. A statistically significant association between PDC grades with N and TNM stages suggested that PDC grades are potential predictive parameters for invasive and metastatic risks in CRC.

## Introduction

Colorectal carcinoma (CRC) ranks third in terms of incidence but second in terms of mortality globally. Meanwhile, CRC is the most common cancer in males and the second most common cancer after breast in females in Malaysia. The CRC incidence was most prevalent among Chinese, followed by Malay and Indian males (1). Furthermore, CRC incidence reportedly increased with age. Precisely, generational shifts were reported in most age-period-cohort analyses and associated with eating habits, obesity and lifestyle. In contrast, CRC mortality has declined in developed nations due to better cancer care and treatment methods, thus improving the chances of survival. Screening and early detection programmes have been proven effective in the USA and Japan since the 1990s (2, 3). Currently, several screening and diagnostic methods exist for CRC patients in Malaysia. An early screening method includes an immunochemical faecal occult blood test (IFOBT), preferable for the population aged 50 years old and above with average risk. Alternatively, colonoscopy is a method of choice for the moderate and high-risk groups (4).

In 2008, a new grading system based on poorly differentiated clusters (PDCs) was introduced (5), demonstrating a high prognostic value for CRCs. The PDCs are made of at least five tumour cells with no gland formation compared to tumour budding (TB) foci, consisting of less than five tumour cells. Resultantly, PDCs are easily detected using haematoxylin and eosin (H & E) staining at the invasive tumour front and in the stroma (6). Contrary to conventional tumour grading methods focusing on the predominant tumour morphology or differentiation, PDC grading emphasises poorly differentiated cancer cells. It was also reported that PDCs, even if focally present, influence the clinical results. Furthermore, low-grade tumour determined using conventional grading methods tend to be high-grade according to the PDC grading. Numerous studies have highlighted that the PDC-based cancer grade is superior to the conventional grade in interobserver agreement and risk classification (7–9). Consequently, it was hypothesised that the PDC grade could be a more reliable option in CRC grading and will be suggested in the upcoming format for CRC reporting (7, 9–12). Nevertheless, further investigations are essential to enhance the prognostic accuracy of PDC grading. Independent of the pathologic tumour, node and metastasis (pTNM) stages, PDC grading was also revealed as a significant predictor of cancer-specific survival (CSS) and recurrence-free survival (RFS) to CRC (7–10, 13).

Various theories on PDC pathogenesis have been developed to obtain more information concerning PDC generation. The successive development of TB into PDC likely stemmed from similar morphologies and originating from the same tumour mass. In vitro studies exhibited that single or aggregates of tumour cells can break from the tumour bulk and maneuver into the desmoplastic extracellular stroma through cohort migration or mesenchymal-amoeboid alteration, triggering the epithelial-mesenchymal transition process (13–18). This finding suggests that PDC may depict TB development as they acquire the capacity for proliferating and aggregating. Thus, PDCs have been closely linked to: i) the up-regulation of Wnt/beta-catenin signaling pathways, such as metalloproteinase (a disintegrin) and L1-cell-adhesion-molecule (L1CAM) (19, 20) or beta-catenin (19–21) and ii) the absence of pro-adhesion proteins, such as cadherin E (21, 22) or claudin (23). Therefore, PDC is potentially involved in tumour spread by invading lymphatic vessels. Moreover, PDC may be essential in determining tumour aggressiveness and predicting tumour behaviour.

The prognostic value of this novel grading system has been verified in multiple research (8, 24). Preoperative biopsy specimen with PDC has been proven viable in predicting nodal metastasis (9). Additionally, this grading system has also been integrated into other tumour such as the breast (25). Despite that, the current clinical practice in Malaysia does not include PDC grading in the histopathology report of CRC cases as this new staging system has yet to be widely accepted. Local pathologists are unfamiliar with this grading system due to a lack of international consensus. Thus, this study findings could provide baseline information on PDCs for CRC cases in a local setting. To the best of our knowledge, this is Malaysia’s first study that analyses PDC in CRC. Further studies and investigations should be conducted in Malaysia to standardise the PDC grading system as part of routine colorectal specimen reporting and subsequently utilised as a prognostic marker for treatment options in CRC patients.

## Methods

This retrospective, cross-sectional study utilised the data of patients diagnosed with CRC who underwent radical colorectal surgery and regional lymphadenectomy in Hospital Selayang, Selangor, from July 2014 until December 2017. The inclusion criteria were CRC cases from resected specimens with a histopathological diagnosis of adenocarcinoma. Meanwhile, the exclusion criteria include mucinous carcinoma with malignant cell clusters in the sizeable mucinous pool, patients with preoperative neoadjuvant chemo and radiotherapy, and several primary cancers such as synchronous and metachronous tumour, local resection and polypectomy. Finally, 47 cases were included based on the correlation study formula, N = [(Zα + Zβ) ÷ C]^2^ + 3, where the calculated sample size was 30 cases.

**Figure 1:**
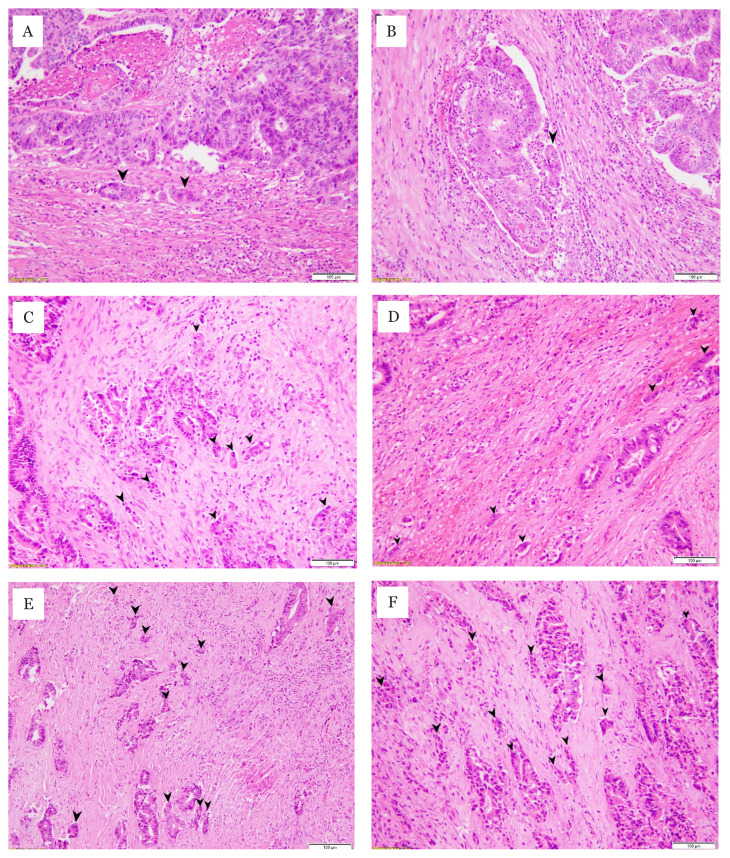
PDCs G1 (A, B), G2 (C, D) and G3 (E, F). (H & E stain, original magnification ×20)

The H & E staining slides of previously diagnosed colorectal adenocarcinoma were retrieved. First, the entire tumour (centre and invasive front) in all tumour-containing slides was subjected to a low-power microscopic examination using a ×4 objective lens and the areas with the greatest PDC density (hot spot) were identified. PDCs were counted under the ×20 microscopic field (with a major axis of 1 mm), with the highest PDC density for grading evaluation. Subsequently, CRC was graded following the PDC clusters: grade 1 (G1) = < 5 PDC clusters, grade 2 (G2) = 5–9 PDC clusters and grade 3 (G3) = ≥ 10 PDC clusters (Figure 1). Two pathologists blinded to the patient’s clinical data or the previous histopathological report performed the scoring independently. A minimum of 90% agreement was accepted, and the discrepancies were resolved by simultaneous reevaluation and discussion.

The results were analysed using a Statistical Package for the Social Sciences (SPSS) for Windows version 25.0 (IBM, USA). The association between PDC grades with demographic and clinicopathological characteristics was analysed using the Pearson’s chi-square test and Fisher’s exact test (for variables with more than 20% having an expected count of less than 5). A *P*-value < 0.05 was considered statistically significant.

## Results

### Distribution of Demographic and Clinicopathological Characteristics of Colorectal Cancer with PDCs

The patient’s ages ranged from 42 years old to 84 years old, with a mean age of 64.36 ± 9.554 years old. Most patients were ≥ 50 years old (93.6%), while the remaining 6.4% were < 50 years old. Furthermore, the majority of the patients were non-Malay (70.2%) and predominantly Chinese (66%), followed by Malay (29.8%). There were more male CRC patients (57.5%) than females (42.5%).

Most CRC patients were of stage T3 (78.7%), followed by stage T2 (17%) and, stages T1 and T4 with equal number patients (2.1%). In addition, the CRC patients were between stages T2 and T4 in terms of tumour invasion; a majority of them were in stage T3 (66%), followed by stage T4 (27.6%) and stage T2 (6.4%). Most cases also recorded lymph node involvement (N1 and N2) (38, 81%). Meanwhile, 24 (51.1%) cases demonstrated lymphovascular and perineural invasions (Table 1).

### Association between Poorly Differentiated Clusters Grades with Demographic and Clinicopathological Characteristics

Out of the 47 CRC cases, seven (14.9%) were graded as G1, 13 (27.7%) were G2 and 27 (57.4%) were G3. There was a significant positive correlation between PDC grades with the nodal stage (N) (*P* = 0.013) and TNM stage (*P* = 0.012). It was observed that the higher the N and TNM stages, the higher the PDC grades. Nevertheless, there was no significant correlation between PDC grades with T stage, lymphovascular invasion, perineural invasion, age, gender and race (Table 2).

## Discussion

Recent research demonstrated that PDCs are strongly associated with lymph node metastasis (11, 12, 26). This current study’s findings have contributed to the percentage. Furthermore, the PDC grading system predicted nodal status with greater sensitivity and specificity than the conventional grading system (8, 9, 27). These findings supported the application of PDC grading in evaluating the risk of lymph node involvement in CRC. The PDC grading is useful in removing low rectal carcinoma when the optimum number of lymph nodes cannot be assessed (27). In addition, PDCs may be clinically valuable in several cases. First, PDC may help identify stage 2 CRC patients with ‘high-risk’ tumour suitable for adjuvant chemotherapy. Secondly, PDC correlates to a higher risk of nodal metastasis in endoscopically resected pT1 CRC, which may influence the decision to undergo radical surgery.

The PDC was a crucial indicator for lymph node involvement in a study conducted on 3,556 pT1 CRC patients (11). Individuals with pT1 CRC who had PDC in their tumour tissue exhibited higher nodal metastases than those without PDC. Thus, PDC is the best predictive parameter for lymph node metastasis risk after the lymphovascular invasion. In the present study, PDC grade assessment could not be completed due to the lack of T1 tumours, which may have led to the insignificant correlation between PDC grade and T stage. Apart from that, PDC grading exhibited high reproducibility compared to lymphovascular invasion due to interobserver variability (7–10). Therefore, PDC grading is potentially more valuable than other prognostic parameters.

The PDC count in the grading of CRC endoscopic biopsies could provide vital details regarding the biological characteristics of the tumour and the anatomical extent of the disease, indicating a higher precision than the conventional tumour grading system. A high PDC count in biopsy samples strongly suggests the unfavourable microscopic features in resection specimens, such as invasive tumour borders, TB, and lymphatic and perineural invasion, all signs of aggressive tumour behaviour (9). Meanwhile, a favourable consensus was achieved amongst observers when evaluating the biopsy of surgical samples using PDC grading compared to the conventional grading system (24). This outcome suggests the clinical significance of the PDC grades in selecting therapeutic care for CRC patients, particularly those with rectal carcinoma. Patients with CRC and a low-grade PDC could be treated with local excision, while those with tumour of high PDC may be suitable for invasive operations, such as anterior resection (28, 29).

Several studies have investigated the effectiveness of PDC grades in classifying CRC patients for prognosis. Research has highlighted PDC grading as an important, independent prognostic factor in CRC (8, 11, 12, 24, 27), where higher grades of PDC (G2–G3) are strongly predictive of short, disease-free survival and disease-specific survival, independent of pTNM stages (6, 23) and other unfavourable microscopic characteristics (7, 8, 10, 11, 24, 27). Furthermore, the survival impacts of PDC grade and TNM stage have been evaluated using multivariate analysis. The TNM stage and PDC grade were independently associated with survival outcomes for recurrence-free survival (RFS) and disease-specific survival (DSS). The survival impacts of the intermediate and severe PDC grades (G2 and G3) were higher than the corresponding categories in the TNM stage (stages II and III) (10).

The current study also showed a significant association between PDC grades and the TNM stage, which could be attributed to the significant association between PDC grades and the N stage. Despite that, other prognostic parameters included in the TNM stage, such as the T stage, revealed insignificant association. As for distant metastasis cases, only one case was included in this study which might not contribute to the significant result. Moreover, higher PDC grades were observed in the higher TNM stage. Therefore, high PDC grades could be associated with higher CRC stages and predict tumour aggressiveness.

There are currently no similar studies published in Malaysia to be used for comparison with this study. Nonetheless, one unpublished study was performed on 129 CRC cases from two institutions and graded using the PDC system, yielding the following results: 73 (56.6%) G1 cases, 33 (25.6%) G2 cases and 23 (17.8%) G3 cases. High PDC grades were significantly associated with lymphovascular invasion, nodal metastasis and high pTNM stage (*P* < 0.05), which partly concurred with the current study findings. However, interobserver agreement analysis was not mentioned (26).

A retrospective study reported the prognostic value of PDC in liver metastasis and the association between PDC grade in the metastatic liver lesion and the primary tumour using a similar grading method. The PDC and TB status in the metastatic liver and the primary tumour were comparable, and the malignant morphological features of the primary tumour were retained in the metastatic liver. Additionally, the PDC grade assessment in the liver using the same method as the primary tumour is a valuable, independent prognostic indicator after liver resection. The G1 PDC prognosis was significantly better than G2 and G3, but there was no significant difference between G2 and G3. Apart from that, G1 PDC has nearly twice as high a 5-year survival rate than G3 (30), indicating the importance of PDC grading in evaluating patients with advanced CRC.

Earlier studies have examined the prognostic significance of PDC grading in various carcinomas, such as Crohns disease-associated small bowel adenocarcinoma, breast carcinoma, and auditory canal carcinoma. A high PDC grade was significantly associated with adverse clinical outcomes (25, 31, 32). Notably, PDC grade in breast and auditory canal carcinomas was associated with a robust interobserver agreement (Kappa values = 0.74 and 0.89, respectively), comparable to CRC (25, 32).

## Conclusion

This study revealed a significant positive correlation between PDC grades with N and TNM stages, which concurred with previous studies. Thus, PDC grading is a potential predictive tool to assess invasive and metastatic risks in CRC. Nevertheless, this grading system deserves continued verification in Malaysia before being implemented into routine diagnostic practice to provide additional, valuable prognostic information for patient management. The independent prognostic significance of the PDC grading should be established in large-scale and multi-institutional studies to ensure the reproducibility and standardisation of the assessment and reporting methods.

## Figures and Tables

**Table 1:** Distribution of demographic and clinicopathological characteristics in CRC cases with PDCs

Demographic and clinicopathological characteristics	No. of cases (%) *n* = 47
Age (years old)	
< 50	3 (6.4)
≥ 50	44 (93.6)
Gender	
Male	27 (57.5)
Female	20 (42.5)
Race	
Malay	14 (29.8)
Non-Malay	31 (70.2)
T stage	
T1	0 (0.0)
T2	3 (6.4)
T3	31 (66.0)
T4	13 (27.6)
N stage	
N0	9 (19.0)
N1	23 (49.0)
N2	15 (32.0)
TNM stage	
Stage 1	1 (2.1)
Stage 2	8 (17.0)
Stage 3	37 (78.7)
Stage 4	1 (2.1)
Lymphovascular invasion	
Negative	23 (48.9)
Positive	24 (51.1)
Perineural invasion	
Negative	23 (48.9)
Positive	24 (51.1)

**Table 2:** Association between grades of PDCs of CRC with demographic and clinicopathological characteristics

Characteristics	Grades of PDC			*P*-value^a^

	G1 (%)	G2 (%)	G3 (%)	
Age (years old)				
< 50	0 (0.0)	1 (33.3)	2 (66.7)	
≥ 50	7 (16.0)	12 (27.2)	25 (56.8)	1.000
Gender				
Male	4 (14.8)	6 (22.2)	17 (63.0)	
Female	3 (15.0)	7 (35.0)	10 (50.0)	0.660
Race				
Malay	1 (7.1)	5 (35.7)	8 (57.2)	
Non-Malay	6 (18.2)	8 (24.2)	19 (57.6)	0.551
T stage				
T1	0 (0.0)	0 (0.0)	0 (0.0)	
T2	1 (33.3)	1 (33.3)	1 (33.3)	
T3	5 (16.1)	8 (25.8)	18 (58.1)	
T4	1 (7.7)	4 (30.8)	8 (61.5)	0.748
N stage				
N0	4 (44.4)	2 (22.2)	3 (33.3)	
N1	3 (13.0)	9 (39.1)	11 (47.8)	
N2	0 (0.0)	2 (13.3)	13 (86.7)	0.013*
TNM stage				
Stage 1	0 (0.0)	1 (100.0)	0 (0.0)	
Stage 2	4 (50.0)	1 (12.5)	3 (37.5)	
Stage 3	3 (8.1)	10 (27.0)	24 (64.9)	
Stage 4	0 (0.0)	1 (100.0)	0 (0.0)	0.012*
Lymphovascular invasion				
Negative	6 (26.1)	7 (30.4)	10 (43.5)	
Positive	1 (4.2)	6 (25)	17 (70.8)	0.075
Perineural invasion				
Negative	5 (21.7)	8 (34.8)	10 (43.5)	
Positive	2 (8.4)	5 (20.8)	17 (70.8)	0.178

Notes:

a Pearson’s chi-square test and Fisher’s exact test;

* statistically significant (*P* < 0.05)
